# Understanding systemic barriers to blood donation for people of South Asian ancestry: A qualitative study

**DOI:** 10.1371/journal.pgph.0005163

**Published:** 2025-09-23

**Authors:** Poojan Joshi, Kelly Holloway

**Affiliations:** Donation Policy and Studies, Canadian Blood ServicesToronto, Ontario, Canada; PLOS: Public Library of Science, UNITED STATES OF AMERICA

## Abstract

We explored how South Asian communities in the Greater Toronto Area (GTA) understand and experience blood donation, systemic barriers to donation, and offer recommendations for blood operators to foster inclusivity and equity in blood donation systems. Using a community-based participatory research approach, we conducted semi-structured interviews with 10 community leaders of South Asian ancestry. Reflexive thematic analysis was used to identify themes related to identity, belonging, and barriers to blood donation, and these themes were understood through the lens of transnationalism. Participants demonstrated the complexity of identity and belonging, and the responsibility of balancing cultural ties with integration into Canada. They faced systemic challenges, including settlement, economic and social challenges, racism and discrimination, and demonstrated resilience in balancing personal and community needs and responsibilities. Blood donation served as a personal commitment and an act of service to society. Key motivations to donate included service to humanity, upholding cultural and spiritual values, and reciprocity. Key barriers included accessibility, language barriers, deferral policies, and lack of tailored outreach and education strategies. Despite these barriers, participants demonstrated their commitment to donation and increasing participation in Canada’s blood donation system. Blood operators can work towards addressing systemic barriers to donation by building relationships with community leaders, adopting culturally sensitive outreach strategies, and improving accessibility. Although limited to community leaders in the GTA, this study provides meaningful insights into the challenges faced by South Asian communities. Further research involving other underrepresented demographics can inform efforts to enhance participation, inclusivity, and equity in blood donation systems.

## 1 Introduction

With a significant increase in immigration and a growing ageing population in Canada, there is a subsequent increase in the demand of blood and blood products [1–3]. In 2021, more than 8.3 million people, or approximately 23 percent of Canada’s total population, were immigrants. Notably, since 2017, South Asians have been the largest group of immigrants to Canada, with most immigrants coming from India and Pakistan [4]. As the demographic landscape changes in Canada, there is a subsequent need to build a sustainable donor population that reflects this diversity. By ensuring that individuals from diverse backgrounds feel represented and supported in the donation process, blood services can encourage broader participation and enhance the accessibility and inclusivity of donation. This is particularly important for improving health outcomes among recipients in these communities, who may require closely matched blood group antigens for successful transfusions and transplantations [5].

In light of this growing demand, it is important to explore the factors influencing blood donation within South Asian communities, including their perspectives and experiences of the barriers to donation. In this paper, we present findings from a qualitative study involving South Asian community leaders in the Greater Toronto Area (GTA). As social scientists in a Canadian blood service, our aim was to investigate how these diverse communities understand and experience blood donation, thereby informing blood operators that rely on voluntary non-remunerated donations. We offer concrete recommendations for addressing systemic barriers to donation, which can enhance organizational awareness and capacity within blood operators. Ultimately, our research seeks to foster an equitable and inclusive approach to blood collection in Canada, building a donor population that truly reflects the diversity of the Canadian population.

Our approach to donation studies is informed by the work of scholars who emphasize the importance of viewing blood operators as playing a critical role in promoting and supporting an inclusive donation experience [6–9]. Rather than seeing communities and identities as static, we recognize that they are dynamic and evolving. This approach encourages blood donation systems to move beyond traditional outreach and engagement methods to adopt strategies that better align with this dynamic. Additionally, we look beyond individual donation behaviours and consider the social, economic, and political contexts within which donors operate. In keeping with this approach, we discuss barriers as potentially systemic, meaning that often a barrier does not exist only at the level of an individual choice, but within social circumstances that can shape what is possible. Understanding how barriers can be systemic means that we can also offer systemic solutions. In taking this approach, we aim to contribute to a more representative, accessible, and responsive donation system.

In order to effectively explore the experiences of South Asian donors, we adopted a transnationalism framework [10,11]. This approach allows us to examine how cultural beliefs, practices, and social networks transcend national boundaries and influence the behaviours and attitudes of immigrants in their new contexts, and in shaping their engagement with blood donation in Canada.

We begin by reviewing the existing literature on blood donation within South Asian communities, noting the limited research on South Asian donors outside of South Asia. We then introduce our theoretical framework of transnationalism, detail our methodology, present our findings and discuss the broader implications for blood operators. We conclude with practical recommendations for blood operators to foster a more inclusive and equitable donation experience.

### 1.1 Blood Collection Structures and Practices in South Asia

Understanding blood collection systems in South Asian countries is important in the context of donation in Canada, where a significant number of immigrants originate from this region [4]. Through our literature review, we gained insights into the broader sociocultural contexts that shape donors’ perceptions and practices regarding blood donation. In defining South Asia for our study, we included countries such as India, Pakistan, Bangladesh, Sri Lanka, Maldives, and Nepal. Each of these countries has a unique blood donation system shaped by distinct cultural, regulatory, and healthcare systems [12–16].

For instance, in India, blood collection is managed by a fragmented system that includes public sector organizations, the Indian Red Cross Society, non-governmental organizations, and commercial entities [12]. Donors within this system are categorized as voluntary, family, or replacement donors [12]. While paid blood donation is illegal under the National Blood Transfusion Services Act [16], there is currently no evidence to confirm whether such practices have been entirely eradicated. Similar blood collection practices are observed in other South Asian countries, including Pakistan [13], Bangladesh [14], and Sri Lanka [15]. These practices often reflect broader sociocultural beliefs regarding blood donation, which in turn influence potential donors’ responses to calls for donations.

Below, we present a brief summary of the current scholarship on South Asian knowledge, attitudes, and practices of blood donation, followed by an overview of the limited research conducted on South Asians living abroad. In our review, a majority of the research on donation has been conducted in India, Pakistan, and Bangladesh, so we will focus our discussion on these countries.

### 1.2 Literature on South Asian communities and blood donation

There is a significant body of research on donation practices within South Asia, focusing on individual factors, such as knowledge, attitudes, and practices (KAPs), and social norms [17–33]. These studies demonstrated prevalent knowledge gaps among the general public about the donation process, alongside fears related to needles, pain, and hygiene, which limited participation in donation in South Asian countries [17–19,21–27,30,32–36]. Cultural values and perceptions both encouraged and deterred donation; for instance, altruism motivated some donors, while concerns about weakness, perceived health risks, and fears of fertility impacts discouraged others [24,33,37,38]. Barriers such as accessibility, like clinic location and hours, and the competing demands on women’s time and energy, in addition to prevalent concerns of anemia, also deterred participation [21,23,37,38]. Additionally, social and caste hierarchies further restricted participation in blood donation [35,37]. Overall, the literature demonstrated a combination of individual, familial, and societal factors influencing blood donation practices within South Asia. Understanding these factors is important, especially as they often migrate alongside individuals to new countries and can shape donation experiences of South Asians living abroad.

There is limited research exploring blood donation practices of South Asians living abroad. An empirical UK-based study outlined different factors influencing donation, and limited reviews and commentaries illustrate sociocultural understandings of blood donation, effective strategies for recruitment and retention of diverse donors, while also exploring individuals’ blood donation KAPs outside of South Asian countries [39–42].

The above-mentioned UK-based study explored perspectives and attitudes toward blood donation among Indians living in North London [40]. Joshi & Meakin (2017) found that participants held various perceptions of blood: universality of blood, as having ownership over their blood, and through cultural symbols such as connecting family and social groups. Furthermore, participants spoke of blood donation as an act of service and as beneficial to others and themselves. Barriers to blood donation included fears, lack of knowledge and awareness, accessibility, and social norms. Lastly, participants emphasized the importance of knowing the blood donors and recipients as they preferred to donate to and receive blood from family and community members rather than strangers [40]. This study sheds light on the combination of cultural beliefs, personal attitudes, and social norms that influence blood donation among South Asians living in the UK.

To our knowledge, similar research focusing on South Asians in Canada remains notably absent. This gap is particularly concerning given the growing South Asian population in Canada and their valuable contributions to the national blood supply. To address this gap, we sought to explore the experiences and perspectives of South Asians in Canada, specifically in the Greater Toronto Area. By examining the motivations and barriers they face in making decisions about donation, we gained valuable insights that may help blood operators better support these communities and improve blood donation initiatives throughout the country.

### 1.3 Migration History & Transnationalism

We use the term “South Asian” broadly, encompassing a range of ethnicities, languages, cultures, and religions [10,43]. We acknowledge that the term “South Asian” can obscure the internal diversity and inequities present within the group and recognize that the experiences of South Asian immigrants vary significantly based on their gender, socioeconomic status, educational background, and religion [10]. This identity is also influenced by historical migration patterns and the ongoing negotiation of cultural identity within the contexts of transnationalism. Understanding these complexities is essential for effectively addressing the needs and experiences of South Asian immigrants, particularly in areas such as blood donation.

South Asian immigration to Canada has been historically influenced by the country’s economic needs and shaped by exclusionary immigration policies [43]. Early policies prioritized European immigrants, and limited South Asian migration primarily to male labourers while restricting the entry of women and children. The introduction of a points-based immigration system in 1967 sought to reduce racial biases and open pathways for skilled immigrants. However, many South Asian professionals continue to encounter significant challenges, as their qualifications go unrecognized in the Canadian job market [10]. Understanding this history and these systemic barriers provides insight into how these experiences shape the economic and social progression of South Asian immigrants in Canada, influencing their identities, social networks, and the environment in which they navigate professional and personal development.

A transnationalism framework allows us to explore the complexities of South Asian immigrants’ experiences and their engagement in social activities, such as blood donation. This framework is informed by the work of Tania Das Gupta on transnationalism, political economy, and the South Asian diaspora in Canada [10,43,44]. Transnationalism describes how migrants maintain connections across borders, fostering social, economic, and cultural ties that shape their identities and community engagement [10].

Migration presents unique challenges for South Asian communities, as they navigate the demands of settling in a new country while maintaining ties to their home countries [10]. These challenges influence their sense of belonging and community involvement, and can play an important role in shaping attitudes toward social participation. This is relevant for blood donation which is often framed as a form of social responsibility.

Negotiating identity is also complex for South Asian immigrants, who have to balance multiple identities, especially when migration has occurred multiple times [10]. Experiences of racism and discrimination can create further challenges, yet many individuals show resilience by preserving their cultural heritage while engaging and progressing within Canadian society. In addition to these social dynamics, economic factors also add complexity. Immigrants often face financial pressures that prioritize work and family obligations over community engagement [10,43]. Our analysis is informed by this work on transnationalism, offering an understanding of how participants navigate donation in the broader context of the challenges faced by South Asian communities.

## 2 Materials and methods

### 2.1 Community-based participatory research

This study was carried out using a community-based participatory research (CBPR) framework which aims to address structural inequities and power dynamics by actively involving community members as leaders and contributors throughout the project [45–47].

We partnered with organizations already collaborating with Canadian Blood Services (CBS), including the Sant Nirankari Mission and Sikh Nation, as well as CBS staff who have long-standing relationships with these communities. The Sant Nirankari Mission is a global spiritual movement that emphasizes service to humanity, and they have successfully organized numerous blood donation drives in partnership with CBS for many years. Blood Donation by Sikh Nation is a group of volunteers dedicated to promoting peace and service in memory of the victims of the 1984 aggression against Sikhs. For the past 25 years, they have led annual blood donation campaigns across Canada in collaboration with CBS.

These partners, alongside CBS staff, formed the Community Advisory Team (CAT), whose role was to provide guidance on research materials, data collection, recruitment strategies, and in interpreting the study’s findings.

### 2.2 Recruitment & data collection

We conducted key informant interviews with community leaders to explore their views and experiences of donation. Participant recruitment and data collection took place from January - March, 2023. Participants were recruited through the professional and personal networks of the research team and community partners, as well as through outreach to community groups, organizations, and via snowball sampling. All participants completed a pre-screening questionnaire to confirm their eligibility for the study. All participants provided verbal consent prior to their participation in the study. This study was approved by the Canadian Blood Services Research Ethics Board.

Inclusion criteria included participants who self-identified as South Asian, were actively involved in their community, and were comfortable in the English language. Semi-structured interviews were held via videoconference or telephone. All interviews were audio-recorded with participants’ verbal consent, and recordings were transcribed by a transcriptionist. Participants were offered a $25 e-gift card for their participation. In the interviews, we asked participants about their community of ethnic ancestry, primary concerns for their communities, their perspectives and experiences of racism, blood and plasma donation, and any recommendations they had for the blood operator. We began the interviews by inviting participants to define their community of ethnic ancestry, drawing on our theoretical framework to understand how they see themselves and their connections to their ethnic ancestry and, self-defined, community. This approach helped us gain insight into their community’s unique needs and challenges. By centering these perspectives, we were able to understand the key informants, their communities, and the broader context within which they might consider blood donation.

Interviews were conducted by KH, Scientist at Canadian Blood Services and Assistant Professor at the University of Toronto, and PJ, Research Assistant at Canadian Blood Services. Dr. Holloway’s work focuses on the social actors in blood systems, and her approach is grounded in qualitative methodologies that consider the political, social, and economic contexts of donor behaviour. Joshi’s shared South Asian background and interest in health equity for underrepresented communities may have facilitated rapport with participants and data analysis through a lens of cultural familiarity. Both KH and PJ conducted interviews for the study. The perspectives and experiences of both interviewers informed interpretation and analysis of this study.

Data was analyzed using reflexive thematic analysis [48,49], and informed by interpretive grounded theory [50]. All interview transcripts were de-identified and uploaded into NVivo, a qualitative data analysis software. For analysis, we first familiarized ourselves with the data by reading through the transcripts, immersing ourselves in the content to gain an understanding of the participants’ perspectives. Next, we generated initial codes by conducting a line-by-line coding of the transcripts in NVivo, identifying key features in the data that addressed barriers to blood donation. After coding, we organized the data into broader themes by grouping similar codes together, which helped identify recurring patterns such as community leadership and barriers to donation. In the fourth step, we reviewed these themes to ensure they accurately captured our data. We revisited the coded extracts to verify consistency and clarity, and refined and merged themes where necessary to ensure that they represented the key barriers and facilitators to blood donation. Before finalizing the themes, we met with the Community Advisory Team (CAT) to gather their input, ensuring that the interpretations resonated with community perspectives and lived experiences. This collaborative input from the CAT helped refine the themes and added depth to our analysis. Finally, while grounding our analysis in the perspectives shared by participants, we engaged with theories of transnationalism [10], reflecting on how this framework could offer an understanding of participants’ experiences and perspectives.

## 3 Results

### 3.1 Participant demographics

We conducted semi-structured interviews with 10 participants who identified as community leaders or active members within their communities of ethnic ancestry. Of the participants, eight identified as men and two as women, with ages ranging from 31 to 80 years (mean age: 53.4), and 70% were in their 50s. Nine participants indicated India as their country of origin, and one participant was from Pakistan. All participants were first-generation immigrants to Canada. Five participants had long-standing partnerships with CBS, two had recently begun collaborating with CBS, two had limited awareness of CBS and no current relationship, and one participant was actively working to build a partnership to organize blood donation clinics in their community.

### 3.2 Overall findings

In our analysis, we found that, for many participants, identity and belonging are fluid and manifest through balancing cultural ties with their roles in Canadian society. Participants also feel a sense of belonging and social responsibility in Canada, while also facing systemic barriers that highlight their differences. This dynamic informs their engagement with blood donation, as they see it as both a personal responsibility and a contribution to society.

Participants’ leadership and advocacy efforts, including community service, social and spiritual engagement, and advocacy, reflects their commitment to thriving in Canada while addressing social and economic challenges. In this context, blood donation serves as an expression of their identity as it bridges their cultural values with contributing to Canadian society. However, systemic barriers, including accessibility, language and cultural disconnects, and deferral criteria, often restrict participation, highlighting the need for minimizing these barriers to improve participation. This results section details these themes, exploring connections between participants’ identities, systemic barriers, and recommendations for fostering inclusivity and equity in blood donation.

### 3.3 Difference, belonging, and shifting definitions of community

Participants’ responses illustrated that definitions of community and identity are fluid and multifaceted. They spoke about their identities as consisting of both specific ethnic and religious affiliations, and broader categories such as global identities. Importantly, participants viewed themselves as part of Canadian society, however, noted that they faced systemic challenges that marked them as different. Most participants did not identify primarily as South Asian, but instead identified as belonging to their home country. One participant specifically disliked the term South Asian as it failed to capture the physical and cultural diversity of the region. Given the complex cultural and political affiliations and rich histories of South Asian countries, labeling participants simply as South Asian does not reflect their identity nor their experiences. Another participant reflected,

…women like me, let’s call us women of colour or immigrant women, we first of all need to kind of find our space in this new community, or this new land that we’ve chosen to immigrate, but then also to kind of find our identity, right, like, because it’s a whole big spectrum. And like I said, I first heard of the term South Asian when I came to Canada; I had no clue what that really was. I was kind of a proud Indian…So, we have these very territorial tags attached to us before we come here, and then I’m glad that you… kind of give that away and you become more of a global citizen where a lot of issues that impact racialized women, or racialized populations actually impact you. So, I stopped thinking of myself as just a Brown person, or just an Indian, and I kind of became part of the bigger conversation [P09].

This participant’s reflection illustrated how her identity changed through migration, to a more connected global identity shaped by her experiences.

Community leadership played an important role in participants’ identities, as they actively engaged in local community and broader Canadian initiatives. Their volunteer work showed their dedication to serving society while also honouring their cultural ties. For example, participants regularly organized community-wide food drives and COVID-19 vaccination clinics. They also took part in religious and cultural gatherings which strengthened their sense of unity and belonging, and created a strong support system within their communities. Lastly, they celebrated cultural events alongside events such as Canada Day, illustrating their connection to both their culture and to Canadian society.

Several participants also engaged in advocacy efforts, including addressing systemic issues such as racism, islamophobia, and advocacy in healthcare and education. For them, this work became a means to both claim space within Canadian society and highlight the systemic challenges they navigate every day.

### 3.4 Engagement with Blood Donation Amidst Community Concerns

Participants’ engagement with blood donation is impacted by the challenges they face as immigrants in Canada. As they navigate their roles and responsibilities within Canadian society, they face various social and economic hurdles that influence how and if they can participate in initiatives like blood donation. Participants spoke of primary concerns in their communities, including the rising cost of living and strains on essential services like healthcare and education, which significantly impacts their communities’ well-being. These concerns illustrate a complex environment within which participants balance the demands of daily life while trying to support larger societal goals (i.e., blood donation).

Several participants discussed Canada’s immigration policies, expressing concern over inadequate planning and support for newcomers. Participants observed that investments in infrastructure, healthcare, and education have not kept up with this growing demand. Limited access to healthcare services and the declining quality of education remain a concern for international students and Canadian citizens. Some participants pointed to the fact that these overlapping pressures mean that activities like blood donation often compete with more immediate needs related to economic stability, health, and family responsibilities.

Additionally, some participants reported experiencing racism and microaggressions, both when they arrived in Canada and years later, as South Asian Canadians, which affected their sense of belonging. Participants recalled incidents tied to cultural expressions, their accents, and their religious identities. They were also aware that these challenges represented a more global pattern of exclusion and discrimination. In the Canadian context, they spoke about the pressure to prove their humanity and social values in response to global events. They explained,

Often when there are things that happen in South Asia…some kind of religious extremism, any kind of such incident like that, I feel like we as South Asians or we as Muslims or we as Hindus, we as Sikhs become, we have to defend ourselves to people in, in Canada...We end up having to become experts, despite not knowing anything ourselves and to be able to have to say that…we are not so backwards, we are not, we’re not what you see in the media, we’re very different from those things. So when those things happen, I often that’s when I guess I, I realize how the world sees us… [P08].

This reflection illustrates the burden of representation faced by many South Asian immigrants, who have to navigate stereotypes while defending and contextualizing incidents to disprove racist Western narratives of extremism and backwardness.

In addition to this, participants also felt a responsibility to prove their contributions to Canadian society:

… having these conversations, you know, working within the community, to let people see the positive side, I think that counteracts what we’re dealing with, right? Because sometimes they’ve never met us, they don’t know that, you know, the misconception is immigrants come here, we take their jobs, we don’t pay taxes, we’re always sending money back home [P10].

and to break down stereotypes. A participant experienced stereotypes at a donation centre:

When I had done a blood drive, one of the ladies who works there actually said to me, why are you here? What are you doing? And I said, well, I’m, you know, like, I run a group, I don’t, I’m not able to donate any more. She was and I said, obviously, I’m trying to get my community to donate blood, oh, I thought your community didn’t do it [P10].

As a response to these challenges, some participants became involved in advocacy efforts, such as anti-racism and anti-Islamophobia initiatives. One participant reflected on their own experiences and advocacy efforts,

Advocacy starts with yourself, and I found so many gaps... And then I realized that, you know, if we don’t advocate for ourselves, really there’s nobody doing it for you. If you’re going to wait for like a political leader or an MP to do it, it’s never going to happen. So, I stopped thinking of myself as just a Brown person, or just an Indian and I kind of became part of the bigger conversation. And really, those dialogues started really early on, I started attending meetings, I started kind of going to my kids’ school, the public library, and I found, like I said, I found big gaps. Nobody was really speaking for us and with us, and that’s how that kind of advocacy thing started for me [P09].

This participant’s shift from a local identity to a broader South Asian identity shows how her immigrant experiences influenced this change. This new “South Asian” identity emerged out of necessity, and served as a way to navigate shared experiences of exclusion and as an avenue for collective action in her new social context.

These advocacy efforts illustrate participants’ commitment to addressing broader social inequalities and barriers, which also extends to their engagement with blood donation. Just as they work to foster inclusion and representation within Canadian society, participants approach blood donation not only as another way to serve and support their communities, but to uphold the responsibility of proving that they belong in Canada by demonstrating these morals and social contributions.

### 3.5 Blood Donation as spiritual connection and community engagement

Participants identified several motivations for blood donation, including social responsibility, cultural values, and personal experiences. Participants described how their family and religious teachings emphasized the importance of helping others and that these values were taught to them from a young age. These teachings framed donation as a commitment to serving humanity.

Participants also emphasized the social nature of blood donation in their reflections, as they often donated as a group, with family, friends, and community members. This social element transformed the act of donating blood from an individual act to a community and social event, which also reinforced a shared culture of giving back. For some participants, blood donation also became a multi-generational activity, as children learned the importance of giving by watching their parents and relatives participate together.

Blood donation also held emotional and symbolic significance for certain participants. Some participants linked their donations to historical events, such as honouring victims of the aggression against Sikhs. Others participated in blood drives during significant times like Thanksgiving to express gratitude for their own health. Finally, personal experiences, such as witnessing loved ones in need of blood transfusions, fostered a sense of reciprocity among participants.

Another motivation was that many participants expressed a strong commitment to blood donation based on their belief in serving humanity, and a sense of social responsibility. In this way, blood donation served as a community-oriented activity, and as moving beyond cultural and national boundaries to represent a commitment to Canadian society. An excellent illustration is that several participants had established long-term partnerships with Canadian Blood Services, and took the initiative to organize community-led blood donation clinics. One participant, who pioneered a blood donation campaign within their community, described that their motivation was driven by spiritual values and a desire to continue donation efforts begun in India. They discussed their experiences and difficulties in organizing blood donation clinics within their community in the 1990s, including meeting logistical requirements by the blood operator. However, they persisted in gathering volunteers and raising awareness of donation within the community.

Other participants also described their experiences and efforts of organizing annual blood donation clinics in their community. These volunteers hosted multiple successful donation clinics each November, overseeing a range of tasks including welcoming donors, distributing snacks, and assisting with language support or screening questionnaires as needed. They noted, however, that logistical issues persist, such as needing to find replacement donors when others could not attend. These challenges highlight the need for ongoing communication and flexibility from the blood operator to support these community-led initiatives.

### 3.6 Barriers to donation in blood systems

We have already pointed to how systemic barriers to donation exist at multiple levels for South Asian communities, including challenges with health systems, immigration, education and cultures, as well as in ideas and systems of oppression that are woven into those institutions. Participants also spoke to systemic barriers that could be addressed by the blood system.

Accessibility was one of the most frequently discussed barriers to donation. Many participants reported that blood donation centres were not conveniently located. They suggested that integrating donation events into community gatherings could make them more accessible. Additionally, participants noted challenges with accessing online systems and childcare, which hindered their ability to donate. Even those who partnered with CBS faced logistical challenges when attempting to bring mobile clinics into their neighbourhoods, often needing to meet attendance quotas and other administrative requirements.

Language barriers also affect accessibility, as many participants who are not fluent in English struggle with screening and donation processes. Without translated materials or bilingual staff, non-English speakers may find it difficult to understand eligibility criteria or communicate their health concerns, leading some to avoid donation. This finding offers support for community-based mobile clinics where community members can provide support to non-English speaking donors, which may enhance their experience and relieve discomforts related to donation.

Deferral criteria posed another significant barrier, as participants referred to travel-related deferrals and health-based ineligibility. These policies can disproportionately affect South Asian communities because of travel to countries that are subject to deferral criteria for malaria risk. Participants said women in their communities have higher rates of anemia, which can impact their eligibility, highlighting how certain health concerns prevalent in South Asian communities intersect with eligibility criteria to limit participation [14,51].

Another barrier was fear related to needles, fainting, and worries over losing strength after blood donation. Cultural perceptions linking physical strength to body weight sometimes intensified these anxieties. Participants noted that education helped alleviate some fears, but initial anxieties regarding donation persisted, suggesting that more culturally sensitive education campaigns are needed to fully address misconceptions and fears in these communities.

In addition, a lack of awareness about the blood operator’s role and the importance of blood and plasma donation is a significant barrier, particularly for those who are less engaged with the blood operator. This lack of awareness highlights a need for targeted outreach to ensure that South Asian communities understand the impact of their contributions and the role of CBS in Canadian healthcare.

Finally, personal commitments also play a role in limiting blood donation participation. Participants frequently balance family obligations, work, and other responsibilities, making it challenging to prioritize donation. Integrating donation events into community gatherings or familiar spaces could help bridge this gap, allowing them to donate while meeting other responsibilities.

Overall, these systemic barriers illustrate the structural and cultural disconnects within the Canadian blood donation system. The layered challenges of immigration, personal and community responsibilities, and systemic barriers reveal a need for more culturally responsive practices. Enhancing accessibility, providing language support, updating deferral policies, and fostering educational outreach tailored to South Asian communities would help create a more inclusive donation environment.

### 3.7 Addressing barriers to donation in blood systems

Participants consistently voiced the need for their challenges to be recognized, highlighting that blood operators should avoid reinforcing systemic barriers already present within healthcare and immigration systems in Canada. By offering accessible donation sites, language support, regularly updating deferral criteria and engaging in targeted educational efforts, blood operators can help reduce misconceptions of donation, making the process feel more approachable to diverse communities.

Promoting a sense of belonging is equally important in overcoming barriers to donation. Participants consistently demonstrated their dedication to community service, with a commitment that extends beyond their ethnic groups to include all Canadians. Blood operators can tap into this broader social responsibility by framing donation as a collective contribution to Canadian society rather than an individual effort. Engaging with community leaders and participating in open, culturally respectful dialogues is important in raising awareness of blood donation and the blood operator, building trust, and encouraging participation.

## 4 Discussion

This qualitative study with community leaders in the GTA aimed to investigate how South Asian communities understand and experience blood donation in Canada. We also engage with frameworks of transnationalism as a way to understand the socio-political dimensions of experiences of South Asian communities in Canada.

Our findings point to the intersections of identity, belonging, and resilience among South Asian communities in Canada and the ways in which donation is intertwined in this complex narrative. For participants, identity and belonging are fluid; they feel a dual sense of belonging and social responsibility in Canada while also encountering systemic barriers that highlight their differences. This dynamic informs their engagement with blood donation, seeing it as both a personal responsibility and a contribution to society. Barriers to donation exist at multiple levels, from the blood operator’s policies to broader struggles with immigration, healthcare, education, settlement and racism.

Our study also highlights the specific challenges faced by South Asian communities living outside South Asia in participating in activities such as blood donation, shaped by their sociocultural understandings of blood and donation. These findings resonate with Joshi & Meakin’s (2017) study of Indians living in North London, in that blood donation was seen as an act of service and as beneficial to themselves and to others. Their study highlighted barriers to donation including fears and concerns related to donation, lack of awareness for the need of blood, especially the knowledge that patients often require regular transfusion to manage chronic illness, accessibility, and social norms [40]. Lastly, participants in their study reported that donation was not something they were regularly exposed to, and that advertisements about donation were infrequent and often forgotten.

Our study contributes to this limited literature by adding another layer of understanding of how transnationalism and the complexity of identity shape the significance of blood donation for South Asian communities. South Asian communities may see donation as a way to bridge the complexities of belonging and identity, and thus it carries meaning that is related to feelings of belonging. Through blood donation, participants advocate for the inclusion and recognition of underrepresented communities in Canada’s healthcare system. Their involvement represents the contributions that immigrant and racialized communities make toward collective well-being, challenging any assumptions that cultural difference may inhibit their engagement [6,8]. By consistently organizing blood donation events and encouraging others in their communities to participate, they demonstrate that cultural identity and social engagement are interconnected. This advocacy and contribution becomes a way to assert not only their values but also their space within Canadian society.

When participants faced barriers to donation, it became part of the broader context of their struggle with identity and belonging in Canada. Participants navigated competing pressures tied to their identities, their roles within family and community, and the broader Canadian context, where systemic inequities exist. These challenges informed the context within which participants engaged in blood donation. For them, blood donation was not just a social duty but an activity that required navigating logistical, cultural, and structural barriers within the existing healthcare and donation system. Participants encountered systemic barriers such as inaccessibility, language barriers, deferral policies, and the lack of tailored community outreach. Each of these obstacles add to the challenges they already take on as immigrants, making participation in blood donation yet another area where they have to work to adapt the blood operator’s processes to better align with their needs. Attention to this context has important implications for how blood operators could address barriers to donation.

### 4.1 Recommendations for blood operators

By looking beyond individual barriers to consider the social, economic, and political contexts within which donors operate [6–9], our study was able to identify recommendations for blood operators that can address systemic barriers to donation (see Table 1).

**Table 1:** Recommendations and strategies for blood operators.

Recommendation Actions	Action-specific Strategies
Community Engagement	• Collaborate with South Asian community leaders to guide outreach efforts • Attend and support key community events and initiatives • Build and maintain long-term partnerships and trust
Culturally relevant information	• Co-create materials with South Asian communities featuring donors/recipients • Describe and clarify donation process, deferrals, and safety and recovery information • Promote donation during culturally meaningful times and in familiar spaces
Minimize practical barriers	• Offer mobile donation clinics when possible and ensure organizational capacity to support mobile clinics • Allocate time and space to address donor questions and concerns • Approach deferrals with cultural sensitivity, care, and appreciation

#### 5 Conclusion

While our study provides valuable insights into the sociocultural understandings of blood donation and the barriers faced by South Asian communities in Canada, we identified some limitations. While participants in this study were primarily from India (with one participant from Pakistan), we recognize that South Asian communities are diverse and include a range of countries, languages, religions, and cultures. As such, the findings from this study should not be generalized to all South Asian communities. Specifically, as the focus of this study was on community leaders in the GTA, our findings may not fully capture the diversity of experiences across different South Asian demographics. However, these leaders have been involved in their communities for many years and are well-positioned to speak to the collective perspectives and experiences of donation and the barriers faced. In this way, they serve as informed representatives of their communities, and provide a comprehensive understanding of the sociocultural and systemic factors influencing blood donation practices. Lastly, our focus on systemic barriers in the Canadian context highlights important challenges but does not address variations in healthcare systems and donation practices in other contexts**.** We recognize these limitations of the current phase of this research. In the next phase of our research, we aim to address these gaps by expanding our focus to include a broader and more diverse sample of participants and explore the prevalence of barriers to donation as well as perspectives on recommendations to address barriers. Lastly, participants in this study were required to be comfortable participating in English. This criteria was based on our limited capacity to conduct and translate interviews in multiple languages. As such, this study may not fully reflect the perspectives of individuals within South Asian communities who are not fluent in English, which may include newcomers and older adults. Importantly, this limitation reflects and may reinforce the language barrier identified in this study. We recognize this important gap and are working to secure funding to include interviews in multiple languages in future research.

Eliminating barriers to donation requires recognizing and accommodating the diverse identities and motivations of donors. By fostering and enhancing a sense of belonging, blood operators can remove barriers and create a more equitable and inclusive donation environment. This approach will not only increase participation but also contribute to a more representative and sustainable blood supply in Canada.
